# The mortality risk of deferring optimal medical therapy in heart failure: a systematic comparison against norms for surgical consent and patient information leaflets

**DOI:** 10.1002/ejhf.838

**Published:** 2017-06-08

**Authors:** Sameer Zaman, Saman S. Zaman, Timothy Scholtes, Matthew J. Shun‐Shin, Carla M. Plymen, Darrel P. Francis, Graham D. Cole

**Affiliations:** ^1^ International Centre for Circulatory Health, National Heart and Lung Institute Imperial College London London UK

**Keywords:** Heart failure, ACE inhibitor, Beta‐blocker, Aldosterone antagonist, Meta‐analysis, Mortality

## Abstract

**Aims:**

The prescription of optimal medical therapy for heart failure is often delayed despite compelling evidence of a reduction in mortality. We calculated the absolute risk resulting from delayed prescription of therapy. For comparison, we established the threshold applied by clinicians when discussing the risk for death associated with an intervention, and the threshold used in official patient information leaflets.

**Methods and results:**

We undertook a meta‐analysis of randomized controlled trials to calculate the excess mortality caused by deferral of medical therapy for 1 year. Risk ratios for angiotensin‐converting enzyme inhibitors, beta‐blockers and aldosterone antagonists were 0.80, 0.73 and 0.77, respectively. In patients who might achieve a 1‐year survival rate of 90% if treated, a 1‐year deferral of treatment reduced survival to 78% (i.e. an annual absolute increase in mortality of 12 in 100 patients). This corresponds to an additional absolute mortality risk per month of 1%. A survey of clinicians carried out to establish the risk threshold at which they would obtain written consent showed the majority (85%) sought written consent for interventions associated with a 12‐fold lower mortality risk: one in 100 patients. A systematic review of UK patient information leaflets to establish the magnitude of risk considered sufficient to be stated explicitly showed that leaflets begin to mention death at a ∼18 000‐fold lower mortality risk of just 0.0007 in 100 patients.

**Conclusions:**

Deferring heart failure treatment for 1 year carries far greater risk than the level at which most doctors seek written consent, and 18 000 times more risk than the level at which patient information leaflets begin to mention death.

## Introduction

Effective therapies for heart failure exist, but audit reveals their under‐utilization^1^, ^2^ and a failure to up‐titrate to doses shown to have benefit in randomized clinical trials. The interaction between the doctor and patient provides the crucial opportunity to impart balanced information on the risks and benefits of a treatment.^3^ Patients who are not offered or who do not accept a prescription or up‐titration may wait for considerable time – perhaps 6 months or 1 year – before they are given an opportunity to revisit their decision.

Previous work shows that clinicians engage in asymmetrical behaviour when discussing medical therapy for heart failure.^4^ They assiduously discuss potential ‘adverse effects’ of medical therapy, even when such effects arise no more commonly than in the placebo arm.^5^ In contrast, few clinicians report explicitly communicating the magnitude of benefit of medical therapy that is most tangible to patients: an increase in lifespan.^4^ Calculating lifespan increase precisely is difficult because, for economic, logistical and ethical reasons, randomized controlled trials (RCTs) terminate when prespecified criteria are met rather than following an entire cohort until all have died. However, the expected absolute risk reduction can be calculated if untreated survival and effect size are known. This may have additional relevance to patients and their clinicians as it represents the risk to which the patient is exposed when medical therapy is deferred.

Discussing absolute risk with patients for any health care decision is only relevant if clinicians regard the risk as sufficiently high to warrant its discussion with patients. If the risk of death is very small, doctors may wish to avoid burdening their patients with a very remote possibility. When the risk is higher, a patient might reasonably expect to be warned. It is not known whether, as a group, clinicians have any consistent threshold for mentioning the possibility of death when discussing an intervention with a patient, and, if so, where this threshold lies.

In this study, we calculated, by meta‐analysing the cumulative RCT experience, the absolute risk of deferring heart failure therapy for 1 year. We surveyed clinicians to establish whether their perceptions of this risk are accurate, and whether the risk exceeds their thresholds for mentioning a risk for death during decision making with patients. For comparison, we reviewed official UK National Health Service (NHS) patient information leaflets for common interventions in order to establish what levels of risk for death are deemed sufficiently high to warrant discussion.

## Methods

Our study involves three components. First, we conducted a meta‐analysis to estimate the respective effect sizes of the use of angiotensin‐converting enzyme (ACE) inhibitors, beta‐blockers and aldosterone antagonists to calculate the levels of risk associated with deferring therapy for 1 year. Second, we surveyed clinicians treating heart failure patients to establish: (i) whether their estimates of the risk arising from deferring heart failure therapy are accurate, and (ii) whether there is agreement on the level of risk for death that is considered sufficient to warrant discussion with patients. Third, we reviewed NHS patient information leaflets to establish the levels of risk deemed sufficient to warrant their presentation in official documentation.

### Meta‐analysis

#### Search strategy

We carried out a meta‐analysis of the three major drug classes used in chronic systolic heart failure. We identified the most recent meta‐analysis for each drug class^6^, ^7^, ^8^ that calculated effects on all‐cause mortality in eligible RCTs. We repeated the search from each meta‐analysis in the MEDLINE, Cochrane and EMBASE databases for the 6 months preceding the publication date of the previous meta‐analysis to ensure that no new RCTs had been published. The search strings were: (i) *ACE inhibitors*: PubMed search: (Heart failure [Title/abstract] OR congestive [Title/Abstract]) AND angiotensin‐converting enzyme (inhibitor OR inhibition OR blocker OR blockade) OR ACE (inhibitor OR inhibition OR blocker OR blockade) OR captopril OR enalapril OR lisinopril OR ramipril OR quinapril; filters: randomized controlled trial; publication date from 1 January 1994 to 1 January 2016; humans; (ii) *beta‐blockers*: PubMed search: (Heart failure [Title/abstract] OR congestive [Title/Abstract]) AND (adrenergic beta‐antagonist OR beta‐adrenergic OR beta‐blocker OR beta‐blockade OR carvedilol OR metoprolol OR bisoprolol OR bucindolol OR nebivolol); filters: randomized controlled trial; publication date from 1 April 2012 to 1 January 2016; humans; (iii) *aldosterone antagonists*: PubMed search: (Heart failure [Title/abstract] OR congestive [Title/abstract]) AND aldosterone receptor (antagonist OR blocker OR blockade OR blocking agent) OR canrenoate OR canrenone OR canrenoic acid OR spironolactone OR eplerenone OR RN 52‐01‐ OR RN 107724‐20‐9 OR Aldactone OR Inspra; filters: randomized controlled trial; publication date from 1 January 2008 to 1 January 2016; humans.

#### Inclusion and exclusion criteria

Randomized controlled trials studying chronic heart failure with reduced ejection fraction were included, as long as they utilized a placebo‐controlled arm rather than non‐placebo ‘usual care’. Any RCTs specifically investigating heart failure with preserved ejection fraction or acute heart failure in specific scenarios were excluded from our analyses.

#### Data abstraction

Manuscripts of included RCTs were independently reviewed by two authors to confirm sample size, numbers of deaths in the control and treatment arms, and follow‐up period. If manuscripts were unavailable for an RCT, we included data for that RCT quoted in previous meta‐analyses.

### Statistical methods

We conducted random‐effects meta‐analyses of RCTs for all three drug classes. The primary endpoint was all‐cause mortality. Statistical analysis was undertaken in R (R Foundation for Statistical Computing, Vienna, Austria) using the metafor package^9^ and figures were prepared using ggplot2.^10^

#### Calculation of additional risk arising from deferral

We reviewed data for annual mortality in contemporary registries of heart failure^1^, ^11^, ^12^, ^13^, ^14^, ^15^ in order to utilize appropriate annual mortality profiles. Using the effect size from our meta‐analysis, we calculated the mortality risk that would arise as a result of the deferral of treatment for 1 year for each of these hypothetical baseline mortality profiles using the formula: mortality attributable to deferral = (mortality without deferral/risk ratio of the intervention) − mortality without deferral.

We assumed the effect size would be constant over the time period studied. The mortality attributable to deferral is dependent on the baseline risk of the population, which can vary widely in heart failure.

One way to assess survival in a heart failure population treated with all three agents is to consider the treatment arm of a contemporary aldosterone antagonist RCT, in which the vast majority of patients were also treated with ACE inhibitors and beta‐blockers. This shows treated 1‐year survival of 93%.^16^ Registries often indicate similar or slightly worse 1‐year survival rates, such as 70.4%,^11^ 74%,^12^ 88%,^13^ 92.8%,^14^ 93.6%^1^ and 94.1%,^15^ We chose a treated 1‐year survival rate of 90%, which is better than in many registries,^1^, ^11^, ^12^, ^13^ and close to optimal therapy in low‐risk patients taking part in a contemporary RCT.^16^ Results for treated 1‐year survival rates of 80%, 85% and 95% are provided in the supplementary material online, *Table* S1.

### Survey of clinicians

Unwarned clinical doctors were approached during teaching sessions, lectures or in person and invited to answer a series of questions. Participants were told the questions were part of a research project and were asked to provide verbal consent. Responses were anonymous and no patients were involved; therefore verbal consent was considered proportionate and reasonable. In addition, participants' willingness to fill in the survey proforma voluntarily was taken to indicate consent. Guidance indicated that research ethics committee approval is not required for such a study. The project was supported by management at our institution.

The questions were:

- What level of absolute risk for death from a clinical decision makes that risk worth mentioning?
- Your patient with heart failure is not prescribed an ACE inhibitor. By how much will their absolute risk of death over 1 year be increased?
- Your patient with heart failure is not prescribed a beta‐blocker. By how much will their absolute risk of death over 1 year be increased?
- Your patient with heart failure is not prescribed an aldosterone antagonist. By how much will their absolute risk of death over 1 year be increased?

For each of these questions, clinicians were able to choose from a series of options (i.e. 20%, 10%, 5%, 4%, 3%, 2%, 1%, 0.1% and 0.01%).

### Review of patient information

The NHS website (NHS Choices) includes a centralized patient information leaflet repository.^17^ We reviewed each leaflet for any diagnostic or therapeutic intervention in which risk was expressed numerically, as either a percentage or fraction. We recorded the intervention described and the numerical denominator and numerator provided. Where a range was provided, we used the mid‐point. Where a risk was quoted as ‘less than’ or ‘<’, we recorded this threshold. The numerator and denominator were standardized to a ‘1 in X’ format for ranking and comparison.

## Results

### Part 1: meta‐analysis

We identified 70 RCTs for inclusion (38 studying ACE inhibitors, 21 studying beta‐blockers, 11 studying aldosterone antagonists). The characteristics of the RCTs used in this analysis are shown in the supplementary material online, *Tables* S2–S4. References for the RCTs used in this analysis are provided in the supplementary material online, *Appendix* S1.

#### ACE inhibitors

**Figure 1:**
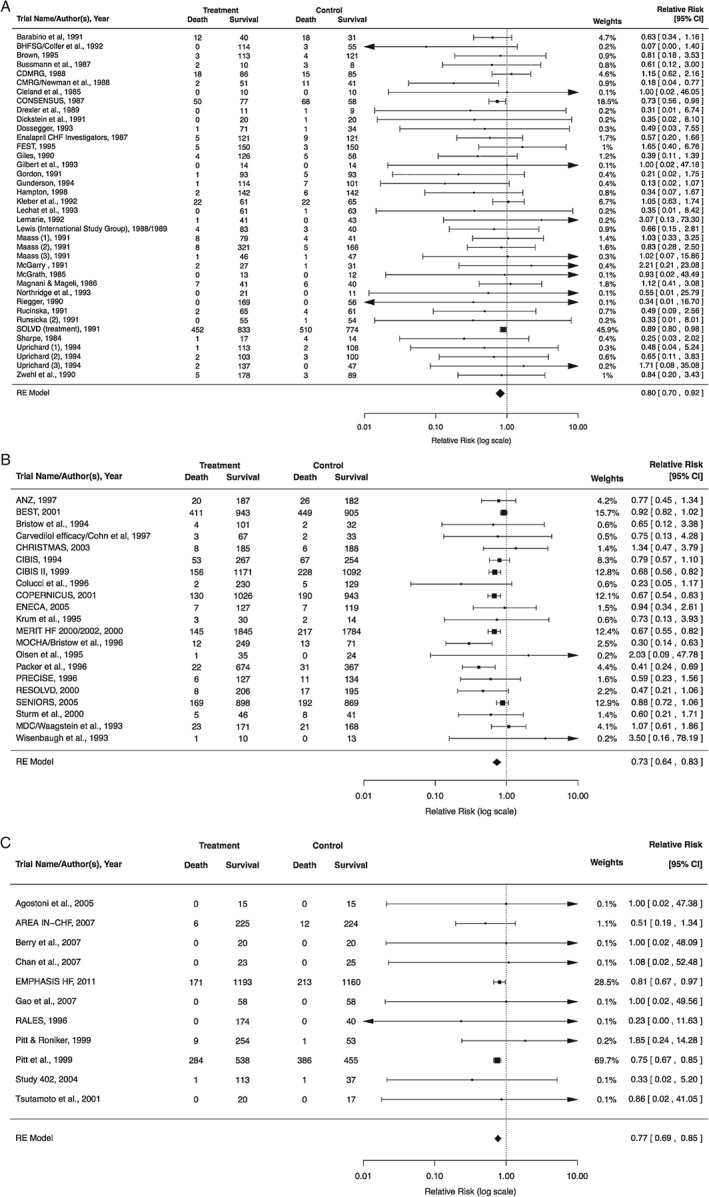
Forest plots for randomized controlled trials (RCTs) of (A) angiotensin‐converting enzyme inhibitors, (B) beta‐blockers and (C) aldosterone antagonists. (See Tables S2–S4, online, for characteristics of RCTs included in these meta‐analyses and Appendix S1, online, for references for all RCTs included.) CI, confidence interval.

The analysis included 38 RCTs. There was a significant reduction in mortality with ACE inhibitors [risk ratio (RR) 0.80, 95% confidence interval (CI) 0.70–0.92; *P* = 0.002] (*Figure* 1 *A*). There was no evidence of heterogeneity (*I* ^2^ = 4.7%; *P* = 0.91).

#### Beta‐blockers

The analysis included 21 RCTs. There was a significant reduction in mortality with beta‐blocker therapy (RR 0.73, 95% CI 0.64–0.83; P < 0.001) (Figure 1 B). There was evidence of moderate heterogeneity (I ^2^ = 43.9%; P = 0.02).

#### Aldosterone antagonists

The analysis included 11 RCTs. There was a significant reduction in mortality with aldosterone antagonist therapy (RR 0.77, 95% CI 0.69–0.85; P < 0.001) (Figure 1 C). There was no evidence of heterogeneity (I ^2^ = 0.0%; P = 0.99).

#### Mortality arising from deferral of therapy for 1 year

**Figure 2:**
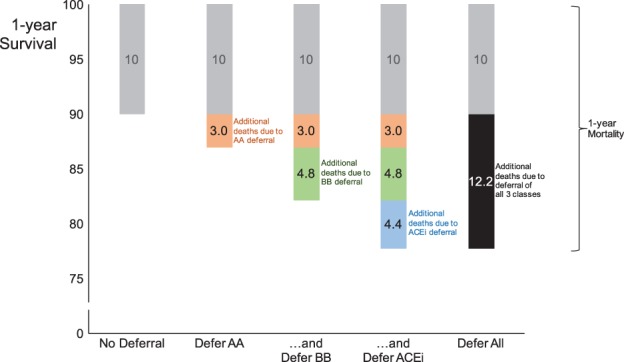
Absolute mortality arising from 1‐year deferral of therapy in a low‐risk patient (90% survival if treated with all three classes of drug). The vertical axis represents the proportion of patients alive at 1 year. The grey bar shows the 10% of patients who will die even if treated with all three drug classes. The orange bar shows the additional 3.0% who will die before 1 year if an aldosterone antagonist (AA) is deferred. The green bar shows the further additional 4.8% who will die before 1 year if a beta‐blocker (BB) is deferred. The blue bar shows the additional 4.4% who will die before 1 year if an angiotensin‐converting enzyme inhibitor (ACEi) is deferred. In total, deferral of all three classes of therapy carries a 12.2% risk for death before 1 year, even for a low‐risk patient.

Figure 2 shows individual increments in mortality caused by deferring the initiation of each of the three drug classes for 1 year, as well as the combined effect of omitting all three. This is calculated from a 1‐year survival of 90% when the patient is treated with all three drug classes. Deferral of an aldosterone antagonist for 1 year (in a patient otherwise fully treated with an ACE inhibitor and beta‐blocker) has a mortality risk of 3.0 in 100. Deferral of a beta‐blocker (in a patient treated with an ACE inhibitor) has a mortality risk of 4.8 in 100. Deferral of an ACE inhibitor antagonist has a mortality risk of 4.4 in 100. Deferral of all three classes of therapy has a combined mortality risk of 12.2 in 100. In Table S1 (online), we provide calculations of the risk arising from deferral of therapy in patients with different treated 1‐year survival rates of 80%, 85% and 95%, respectively.

### Survey of clinicians

Of 233 clinicians invited to answer the questions, 205 accepted, a response rate of 88%.

#### Clinicians' estimation of absolute risk caused by deferring heart failure therapy

**Figure 3:**
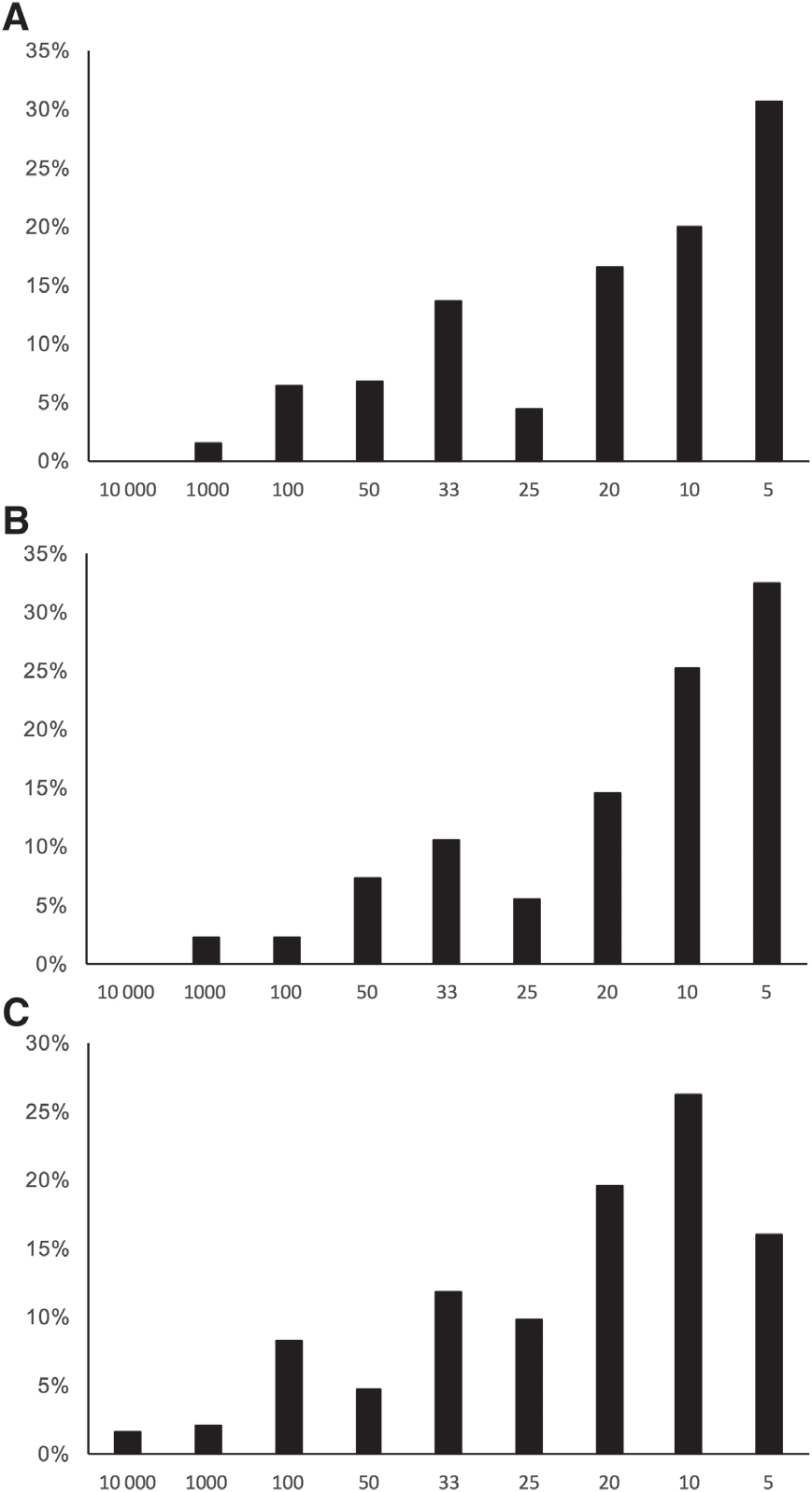
Distribution of clinicians' estimates of absolute risk in a typical heart failure patient associated with deferral of therapy for 1 year with (A) angiotensin‐converting enzyme inhibitors, (B) beta‐blockers and (C) aldosterone antagonists. Numbers on the horizontal axis refer to categories of answer for risk (one in…). Bars represent the proportions of survey respondents choosing that option.

The distribution of clinicians' estimates of the mortality risk arising from 1‐year of deferral of each drug class is shown in *Figure* 3. Although there was a wide spread of estimates, the modal choice was one in five for ACE inhibitors and beta‐blockers, and one in 10 for aldosterone antagonists. These correspond to an absolute mortality increment of between 10 and 20 extra deaths per 100 patients, which is larger than calculated from RCT data, even in patients with treated 1‐year survival of only 80% (*Table* S1, online). Clinicians appreciate and may even overestimate the absolute risk arising from the deferral of medical therapy.

#### Clinicians' threshold for including risk for death when taking written consent

**Figure 4:**
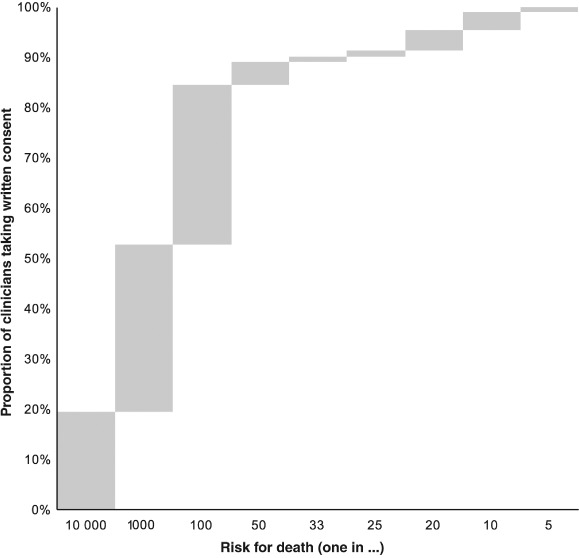
Thresholds at which clinicians discussing a clinical decision will include the risk for death. Responses are arranged from those with the lowest threshold (one in 10 000) to those with the highest threshold (one in five). Bars indicate the proportions of responses in each category. Upper edges of bars show the cumulative proportions of clinicians who will mention death at that level of risk or lower.

Clinicians were asked to indicate the level of risk of death associated with a clinical decision at which they would obtain consent in writing. The distribution of responses is shown in *Figure* 4. Overall, 20% of clinicians obtain written consent for a risk for death as low as one in 10 000. A further 33% (53% in total) take written consent for a risk for death of one in 1000 or less. A further 32% (85% in total) take written consent for a risk for death of one in 100 or less. Only 15% of clinicians would not mention death when taking written consent until the risk was greater than one in 100.

The thresholds at which individual clinicians include reference to death when taking verbal consent for a clinical decision were highly correlated with their behaviour when taking written consent (Spearman's rho = 0.69, *P* < 0.001).

### Systematic review of patient information leaflets that include numerical risks

In the centralized NHS patient information leaflet repository,^17^ we identified 89 leaflets that are designed to help patients make decisions about a test or a treatment, of which 49 quantified a total of 149 risks numerically.

Of the risks described, 13 (8.7%) referred to death or a combination of death and another complication. The risk of death quoted varied from one in 28.6 (death in the first year after pancreas transplant) to one in 150 000 (death from general anaesthesia). The median risk was one in 300 (interquartile range: one in 75 to one in 1000). The risk of death in these patient information leaflets was usually lower than is associated with the deferral of treatment with any individual drug class in heart failure patients with 1‐year treated survival of 90%. Every leaflet quoted a risk of death that was lower than the risk arising from the deferral of ACE inhibitor therapy for 1 year (one in 22) or deferral of beta‐blocker therapy for 1 year (one in 21). Twelve of 13 (92%) leaflets quoted a risk of death that was lower than the risk arising from the deferral of aldosterone antagonist therapy for 1 year (one in 33). Every leaflet quoted a risk of death lower than the risk associated with the deferral of medical therapy with all three drug classes in combination for 1 year (one in eight).

## Discussion

In this study, we show that when medical therapy for heart failure is deferred even for 1 month in a low‐risk patient, the absolute increase in risk of death (∼1%) substantially exceeds the threshold at which most clinicians would take written consent for risk of death arising from a procedural intervention. As *Table* 1 shows, it also massively exceeds the risk for death that is considered sufficient to warrant inclusion in patient information leaflets.

**Table 1:** Comparison of risk for death arising from deferral of medical therapy for heart failure for 1 year (in a patient with 1‐year survival of 90% if treated with all three drug classes) with risk of death mentioned in NHS patient information leaflets. Even in a low‐risk patient, the deferral of any class of treatment involves higher risk than that cited in almost all patient information leaflets that refer to risk for death

Treatment decision	Risk of death:
	*1 in…*
All three drug classes for heart failure: 1‐year deferral	8
Beta‐blocker for heart failure: 1‐year deferral	21
ACE inhibitor for heart failure: 1‐year deferral	22
Pancreas transplant (first year)	29
Aldosterone antagonist for heart failure: 1‐year deferral	33
Aortic valve replacement	50
Gastrectomy (for cancer)	50
Coronary angioplasty	100
Carotid endarterectomy	100
Gastrectomy (for obesity)	100
Spinal stenosis surgery	300
Lumbar decompression surgery	700
Bariatric surgery	1000
Weight loss surgery	1000
Transurethral resection of prostate	1000
Anaesthesia (all types)	100 000
General anaesthesia	150 000

ACE, angiotensin‐converting enzyme.

Why is the risk of deferring effective medical therapy for a patient with heart failure approached differently from procedural risk? One explanation may be that, for a procedure, the risk is concentrated in a shorter time period. Once the decision to undertake a procedure has been made, it cannot usually be reversed. In contrast, a decision to defer prescribing a tablet may in theory be revised, although our experience is that patients risk either being left untreated or being treated with smaller doses than have been shown to be beneficial in RCTs. If additional repeat appointments for patients not taking life‐prolonging therapy led reliably to reconsideration or up‐titration, they might be a surprisingly effective intervention. For the minority of patients who refuse therapy, one way of allowing them to reverse their decision independently may be a prescription from the initial consultation, which can be used to embark on therapy without the need for further consultation. This approach has been shown to be of value in other scenarios.^18^

### Cause of death: disease or deferral?

Another explanation for differing attitudes to risk may be that clinicians (and perhaps patients) view risk associated with an intervention differently from risk arising from a lack of intervention. When a procedural complication arises, causality is usually clear. When it arises after the deferral of an intervention, it is impossible to know for that individual patient whether it was caused by the deferral of effective therapy or would have happened regardless due to the condition itself.

However, in a given population, it is possible to calculate the chance that a death is attributable to deferral. In patients with treated 1‐year survival of 90%, the 10% mortality risk increases to 12.8% with the deferral of an aldosterone antagonist. It follows that 23% (2.8 of the 12.8 percentage units) of deaths over the first year are attributable to the deferral of aldosterone antagonist treatment. In a similar way, in patients with treated 1‐year survival of 90% in which both aldosterone antagonist and beta‐blocker treatment are deferred, 44% (7.8 of 17.8) of deaths arise from deferral. In patients with a treated 1‐year survival of 90% in which all three drug classes are deferred, 55% (12.2 of 22.2) of deaths over the first year will be attributable to the deferral of therapy.

We believe the decision on whether to discuss the risk of death with a patient should depend on the magnitude of risk, rather than whether it arises from an intervention or from the deferral of the intervention. Similarly, it is illogical to decide whether a patient should be made explicitly aware of the risk based on whether it occurs in an operating theatre or at home, or whether there is a theoretical option of reversal. We propose that, given the magnitude of risk involved, the early initiation and up‐titration of medical therapy are of critical importance. If patients wish to decline or defer therapy, it may be appropriate to take written consent.

### Harnessing the efficacy of existing therapies

The act of taking written consent for the deferral of treatment may have a hidden therapeutic value if it helps the patient to focus on the implications of his or her decision. Further therapeutic advances in heart failure are likely to be hard‐won: a new class of treatment has required almost 20 000 patient‐years of follow‐up to demonstrate a ∼1% reduction in annual mortality.^19^ To an optimist, the trial's control arm mortality of 19.8% at 27 months reflects the successful application of existing therapies in patients recruited to RCTs. To a pessimist, it warns us that future advances will be time‐consuming and expensive to demonstrate unless they have unprecedented and implausible^20^ effect sizes. With a largely static therapeutic armamentarium, we suggest maximal application of existing RCT‐proven therapies is increasingly important.^21^

### Limitations

Although the manuscripts for the majority of RCTs identified from previous meta‐analyses were reviewed manually, some manuscripts were unavailable. In such cases, the numbers of patients and deaths in control and treatment arms were extracted from abstracts or existing meta‐analyses. Ideally, our meta‐analysis would have used hazard ratios, but these were unavailable and we therefore used risk ratios instead.

One unavoidable limitation is that our calculation is based exclusively on data from RCTs, which provide the only bias‐resistant measurement of therapeutic effect size.^21^ We assume the effect size seen in RCTs holds for patients less likely to be included in RCTs, such as those with more co‐morbidities.^22^ If clinicians perceive an RCT to study an unrepresentative population, this may lead to slow acceptance of a therapy demonstrated to be effective.^23^ If the effect size is reduced in patients not typically recruited to RCTs, the risk of deferral will be smaller than we calculate, but there is no way to experimentally test whether this is the case because only an RCT can reliably measure effect size.

Another unavoidable limitation is that the risk of deferral is a proportion of baseline risk. We chose to base our calculations on a treated 1‐year survival rate of 90%, which is close to that seen in contemporary RCTs^16^ and within the range of mortality seen in registries. Other baseline treated risks (and therefore risks arising from deferral) are of course possible, and in *Table* S1 (online) we provide the same calculations for a series of different baseline risks.

Finally, our participants were a convenience sample of clinicians encountered in meetings, educational sessions and conferences. We did not attempt to stratify or target particular groups of clinicians. However, heart failure is increasingly common^24^ and is encountered by clinicians who are not heart failure specialists.

## Conclusions

There is startling disparity in clinician behaviour in the context of discussing risk arising from procedural interventions compared with the deferral of life‐prolonging therapy in a chronic disease. For procedures with a risk for death of one in 100 or lower, the majority of clinicians report recording the discussion of this in writing. Yet the decision to defer *any* of the classes of medical therapy for a year, even in a patient with low‐risk heart failure, carries a far greater risk for death. Deferral of all three classes of treatment, even in a patient with low‐risk heart failure, carries an absolute mortality risk of around 1% *per month*.

This asymmetry is not because clinicians fail to appreciate the risk of deferring treatment, which, if anything, they may overestimate. However, more formal consideration of deferral as an active choice with implications worthy of written consent may help to focus patients and their clinicians on the importance of maximal early application of life‐saving therapy.

### Funding

MJS‐S (grant no. FS/14/27/30752), DPF (grant no. FS/010/038) and GDC (grant no. FS/12/12/29294) are funded by the British Heart Foundation.

**Conflict of interest:** none declared.

## Supporting information


**Appendix S1.** References for randomized controlled trials included in the meta‐analyses.
**Table S1.** Absolute increase in mortality (extra deaths per 100 patients) arising from deferral of heart failure therapy in patients who would achieve 1‐year survival of 95%, 90%, 85% and 80% with full treatment.
**Table S2.** Randomized controlled trials of angiotensin‐converting enzyme inhibitors included in the meta‐analysis according to intervention, concurrent medication in the control group, mortality in the control and treatment arms, and follow‐up period.
**Table S3.** Randomized controlled trials of beta‐blockers included in the meta‐analysis according to intervention, concurrent medication in the control group, mortality in the control and treatment arms, and follow‐up period.
**Table S4.** Randomized controlled trials of aldosterone antagonists included in the meta‐analysis according to intervention, concurrent medication in the control group, mortality in the control and treatment arms, and follow‐up period.
