# Supplementary material for: The mortality risk of deferring optimal medical therapy in heart failure: a systematic comparison against norms for surgical consent and patient information leaflets
Source: Eur J Heart Fail. 2017 Jun 8;19(11):1401–9. doi: 10.1002/ejhf.838 (PMC5726382; doi:10.1002/ejhf.838)
Supplement: Supplementary file 1 — Appendix S1. References for randomized controlled trials included in the meta‐analyses. Table S1. Absolute increase in mortality (extra deaths per 100 patients) arising from deferral of heart failure therapy in patients who would achieve 1‐year survival of 95%, 90%, 85% and 80% with full treatment. Table S2. Randomized controlled trials of angiotensin‐converting enzyme inhibitors included in the meta‐analysis according to intervention, concurrent medication in the control group, mortality in the control and treatment arms, and follow‐up period. Table S3. Randomized controlled trials of beta‐blockers included in the meta‐analysis according to intervention, concurrent medication in the control group, mortality in the control and treatment arms, and follow‐up period. Table S4. Randomized controlled trials of aldosterone antagonists included in the meta‐analysis according to intervention, concurrent medication in the control group, mortality in the control and treatment arms, and follow‐up period. [file EJHF-19-1401-s001.docx]

# SUPPLEMENTAL MATERIAL

# Online Appendices for:

# The mortality risk of deferring optimal medical therapy for heart failure – a systematic comparison against norms for surgical consent and patient information leaflets

Sameer Zaman MBBS BSc

Saman S Zaman MBBS MRCP

Timothy Scholtes MSci

Matthew J Shun-Shin MRCP

Carla M Plymen BSc(Hons) MRCP MD(Res)

Darrel P Francis MA FRCP

Graham D Cole MA MRCP

^1^ *International Centre for Circulatory Health, National Heart and Lung Institute, Imperial College, London, UK*

**Online Appendix 1: Characteristics of Randomized Control Trials Included in Meta-analyses**

| **Trial Name/Author(s)/Group** | **Year** | **Intervention** | **Control Group Treatments (Where known)** | **N=** | **Deaths** | **Deaths** | **Follow-up** |
| --- | --- | --- | --- | --- | --- | --- | --- |
|  |  |  | **(Majority or %)** |  | **(control)** | **(treatment)** | **(months)** |
| Barabino et al. ^1^ | 1991 | Captopril | Digoxin, diuretics | 101 | 18 | 12 | 6 |
| BHFSG/Colfer et al. ^2^ | 1992 | Benazepril | Digoxin, diuretics | 172 | 3 | 0 | 3 |
| Brown et al. ^3^ | 1995 | Fosinopril | Diuretics | 241 | 4 | 3 | 6 |
| Bussmann et al. ^4^ | 1987 | Captopril | Digoxin, diuretics | 23 | 3 | 2 | 6 |
| CDMRG ^5^ | 1988 | Captopril | Diuretics (3rd arm: Digoxin) | 204 | 15 | 18 | Unclear |
| CMRG/Newman et al. ^6^ | 1988 | Captopril | Digoxin, diuretics | 105 | 11 | 2 | 3 |
| Cleland et al. ^7^ | 1985 | Enalapril | Digoxin, diuretics (95% furosemide) | 20 | 0 | 0 | 2 |
| CONSENSUS ^8^ | 1987 | Enalapril | Digoxin, diuretics, vasodilators | 253 | 68 | 50 | 6.18 |
| Drexler et al. ^9^ | 1989 | Cilazapril | Digoxin, diuretics | 21 | 1 | 0 | 3 |
| Dickstein et al. ^10^ | 1991 | Enalapril | Digoxin and diuretics (100%), anti-arrhythmics | 41 | 1 | 0 | 12 |
| Dossegger et al. ^11^ | 1993 | Cilazapril | Digoxin (>90%), diuretics (60%) | 107 | 1 | 1 | 3 |
| Enalapril CHF Investigators ^12^ | 1987 | Enalapril | Unclear | 256 | 9 | 5 | Unclear |
| FEST ^13^ | 1995 | Fosinopril | Digoxin, diuretics | 308 | 3 | 5 | 3 |
| Giles ^14^ | 1990 | Lisinopril | Unclear | 193 | 5 | 4 | Unclear |
| Gilbert et al. ^15^ | 1993 | Lisinopril | Digoxin, diuretics | 28 | 0 | 0 | 6 |
| Gordon et al. ^16^ | 1991 | Ramipril | Unclear | 192 | 5 | 1 | Unclear |
| Gunderson et al. ^17^ | 1994 | Ramipril | Digoxin, diuretics | 223 | 7 | 1 | 3 |
| Hampton et al. ^18^ | 1998 | Trandolapril | Diuretics | 292 | 6 | 2 | 4 |
| Kleber et al. ^19^ | 1992 | Captopril | Any medication permitted except ACE Inhibitors | 170 | 22 | 22 | 32.4 |
| Lechat et al. ^20^ | 1993 | Perindopril | Digoxin, diuretics | 125 | 1 | 0 | 3 |
| Lemarie ^21^ | 1992 | Ramipril | Unclear | 85 | 0 | 1 | Unclear |
| Lewis (International Study Group) ^22^ | 1988/1989 | Lisinopril | Digoxin, diuretics | 130 | 3 | 4 | 3 |
| Maass (1) ^23^ | 1991 | Ramipril | Digoxin and furosemide (100%) | 132 | 4 | 8 | Unclear |
| Maass (2) ^24^ | 1991 | Ramipril | Unclear | 500 | 5 | 8 | Unclear |
| Maass (3) ^25^ | 1991 | Ramipril | Unclear | 95 | 1 | 1 | Unclear |
| McGarry ^26^ | 1991 | Benazepril | Unclear | 61 | 1 | 2 | Unclear |
| McGrath ^27^ | 1985 | Enalapril | Digoxin and furosemide | 25 | 0 | 0 | 3 |
| Magnani & Mageli ^28^ | 1986 | Captopril | Unclear | 94 | 6 | 7 | 12 |
| Northridge et al. ^29^ | 1993 | Quinalapril | Digoxin, diuretics | 32 | 0 | 0 | 2 |
| Riegger ^30^ | 1990 | Quinalapril | Digoxin and non-potassium sparing diuretics | 225 | 0 | 0 | 3 |
| Rucinska ^31^ | 1991 | Enalapril | Unclear | 132 | 4 | 2 | Unclear |
| Runsicka (2) ^32^ | 1991 | Lisinopril | Unclear | 58 | 0 | 1 | Unclear |
| SOLVD (treatment) ^33^ | 1991 | Enalapril | Digoxin, diuretic (majority), beta blockers (7%) | 2569 | 510 | 452 | 41.4 |
| Sharpe ^34^ | 1984 | Enalapril | Digoxin and diuretics | 36 | 4 | 1 | 3 |
| Uprichard (1) ^35^ | 1994 | Quinalapril | Unclear | 224 | 2 | 1 | 4 |
| Uprichard (2) ^36^ | 1994 | Quinalapril | Unclear | 208 | 3 | 2 | 4.5 |
| Uprichard (3) ^37^ | 1994 | Quinalapril | Unclear | 186 | 0 | 2 | 3 |
| Zwehl et al. ^38^ | 1990 | Lisinopril | Unclear | 275 | 3 | 5 | Unclear |

**Appendix 1 Table 1: Randomized control trials of ACE Inhibitors included in our analysis. See online appendix 3 for references of the randomized control trials included in this meta-analysis.**

|  |  |  |  |  |  |  |  |
| --- | --- | --- | --- | --- | --- | --- | --- |
| **Trial Name/Author(s)/Group** | **Year** | **Intervention** | **Control Group Treatments (where known)** | **N=** | **Deaths** | **Deaths** | **Follow-up** |
|  |  |  | **(Majority or %)** |  | **(control)** | **(treatment)** | **(months)** |
| ANZ ^39^ | 1997 | Carvedilol | ACEI + diuretics (>50%), digoxin (<50%) | 415 | 26 | 20 | 19 |
| BEST ^40^ | 2001 | Bucindolol | ACEI and diuretics and digoxin (>90%) | 2708 | 449 | 411 | 24 |
| Bristow et al. ^41^ | 1994 | Bucindolol | ACEI, diuretics, digoxin | 139 | 2 | 4 | 3 |
| Carvedilol efficacy/Cohn et al. ^42^ | 1997 | Carvedilol | ACEI and diuretics and digoxin | 105 | 2 | 3 | 6 |
| CHRISTMAS ^43^ | 2003 | Carvedilol | ACEI and diuretics (majority) | 387 | 6 | 8 | 6 |
| CIBIS ^44^ | 1994 | Bisoprolol | ACEI (>90%), diuretics (100%) | 641 | 67 | 53 | 22.8 |
| CIBIS II ^45^ | 1999 | Bisoprolol | ACEI and diuretics (majority), diuretics (52%) | 2647 | 228 | 156 | 15.6 |
| Colucci et al. ^46^ | 1996 | Carvedilol | ACEI, diuretics, digoxin | 366 | 5 | 2 | 12 |
| COPERNICUS ^47^ | 2001 | Carvedilol | ACEI/A2RB (99%), diuretics (99%), digoxin >70%) | 2289 | 190 | 130 | 10.4 |
| ENECA ^48^ | 2005 | Nebivolol | ACEI/A2RB and diuretics (90%), digoxin (50%) | 260 | 7 | 7 | 12 |
| Krum et al. ^49^ | 1995 | Carvedilol | ACEI, diuretics, digoxin (majority) | 49 | 2 | 3 | 3.5 |
| MERIT HF 2000/2002 ^50, 51^ | 2000 | Metoprolol | ACEI and diuretics | 3991 | 217 | 145 | 12 |
| MOCHA/Bristow et al. ^52^ | 1996 | Carvedilol | ACEI and diuretics and digoxin (>90%) | 345 | 13 | 12 | 6 |
| Olsen et al. ^53^ | 1995 | Carvedilol | ACEI and diuretics and digoxin (>75%) | 60 | 0 | 1 | 4 |
| Packer et al. ^54^ | 1996 | Carvedilol | ACEIs and diuretics and digoxin (>95%) | 1094 | 31 | 22 | 12 |
| PRECISE ^55^ | 1996 | Carvedilol | ACEI and diuretics and digoxin (>90%) | 278 | 11 | 6 | 6 |
| RESOLVD ^56^ | 2000 | Metoprolol | ACEI/A2RB, diuretics (83%), digoxin (67%) | 426 | 17 | 8 | 6 |
| SENIORS ^57^ | 2005 | Nebivolol | ACEI/A2RB and diuretics, digoxin (40%), AA (30%) | 2128 | 192 | 169 | 21 |
| Sturm et al. ^58^ | 2000 | Atenolol | Enalapril and digoxin (100%), furosemide (>50%) | 100 | 8 | 5 | 24 |
| Waagstein et al. – MDC ^59^ | 1993 | Metoprolol | ACEI and digoxin and diuretics (>75%) | 383 | 21 | 23 | 18 |
| Wisenbaugh et al. ^60^ | 1993 | Nebivolol | Furosemide | 24 | 0 | 1 | 3 |

**Appendix 1 Table 2: Randomized control trials of beta-blockers included in our analysis (ACEI = ACE inhibitor; A2RB = angiotensin-2 receptor blocker; AA = aldosterone antagonist). See online appendix 3 for references of the randomized control trials included in this meta-analysis.**

| **Trial Name/Author(s)/Group** | **Year** | **Intervention** | **Control Group Treatments (where known)** | **N=** | **Deaths** | **Deaths** | **Follow-up** |
| --- | --- | --- | --- | --- | --- | --- | --- |
|  |  |  | **(Majority or %)** |  | **(control)** | **(treatment)** | **(months)** |
| Agostoni et al. ^61^ | 2005 | Spironolactone | ACEI, BB, diuretics | 30 | 0 | 0 | 6 |
| Barr et al. ^62^ | 1995 | Spironolactone | ACEI and diuretics | 42 | 0 | 0 | 2 |
| Berry et al. ^63^ | 2007 | Spironolactone | ACEI/A2RB (100%), diuretics (70%), digoxin (20%) | 40 | 0 | 0 | 3 |
| Boccanelli et al. (AREA IN-CHF) ^64^ | 2007 | Canrenone | ACEI/A2RB and BB (100%), digoxin and diuretics allowed | 467 | 12 | 6 | 12 |
| Chan et al. ^65^ | 2007 | Spironolactone | Candesartan (100%), BB and diuretics (majority) | 48 | 0 | 0 | 12 |
| Gao et al. ^66^ | 2007 | Spironolactone | ACEI, BB, digoxin, loop diuretic | 116 | 0 | 0 | 6 |
| Pitt (RALES) ^67^ | 1996 | Spironolactone | ACEI and loop diuretic, digoxin | 214 | 0 | 0 | 3 |
| Pitt & Roniker ^68^ | 1999 | Eplerenone | Unclear | 317 | 1 | 9 | 3 |
| Pitt et al. (Spiro) ^69^ | 1999 | Spironolactone | ACEI and loop diuretic, digoxin | 1663 | 386 | 284 | 24 |
| Study 402 ^70^ | 2004 | Eplerenone | Unclear | 152 | 1 | 1 | 3 |
| Tsutamoto et al. ^71^ | 2001 | Spironolactone | ACEI and diuretics and digoxin (majority), BB (40%) | 37 | 0 | 0 | 4 |
| Zannad et al. (EMPHASIS HF) ^72^ | 2011 | Eplerenone | ACEI/A2RB and BB | 2737 | 213 | 171 | 21 |

**Appendix 1 Table 3: Randomized control trials of aldosterone antagonists included in our analysis. (ACEI = ACE inhibitor; A2RB = angiotensin-2 receptor blocker; BB = beta-blocker). See online appendix 3 for references of the randomized control trials included in this meta-analysis.**

**Online Appendix 2: Absolute risk of death associated with deferral of medical therapy for heart failure for one year**

| **Absolute percentage increase in mortality arising from 1-year deferral patients with:** | Deferring | Deferring | Deferring |  | Deferring |
| --- | --- | --- | --- | --- | --- |
|  | AA | BB | ACEI |  | AA, BB & ACEi |
|  |  |  |  |  |  |
| **95%** survival when treated with all 3 classes | **1.5** | **2.4** | **2.2** |  | **6.1** |
| **90%** survival when treated with all 3 classes | **3.0** | **4.8** | **4.4** |  | **12.2** |
| **85%** survival when treated with all 3 classes | **4.5** | **7.2** | **6.7** |  | **18.4** |
| **80%** survival when treated with all 3 classes | **6.0** | **9.6** | **8.9** |  | **24.4** |

**Appendix 2 Table 1: Absolute increase in mortality (extra deaths per 100 patients) arising from deferral of heart failure therapy for patients that would have 95%, 90%, 85% and 80% one-year survival with full treatment. (AA = aldosterone antagonist; BB = beta blocker; ACEI = ACE inhibitor)**

**Online Appendix 3: References for randomized control trials included in meta-analyses**

**ACE inhibitor randomized control trials**

1. Barabino, A., Galbariggi, G., Pizzorni, C. & Lotti, G. (1991) Comparative Effects of Long-Term Therapy with Captopril and Ibopamine in Chronic Congestive Heart Failure in Old Patients. *Cardiology.* 78 (3), 243-256.
2. Colfer, H. T., Ribner, H. S., Gradman, A., Hughes, C. V., Kapoor, A. & Laidlaw, J. C. Effects of once-daily benazepril therapy on exercise tolerance and manifestations of chronic congestive heart failure. *American Journal of Cardiology.* 70 (3), 354-358.
3. Brown, E. J., Chew, P. H., MacLean, A., Gelperin, K., Ilgenfritz, J. P. & Blumenthal, M. (1995) Effects of fosinopril on exercise tolerance and clinical deterioration in patients with chronic congestive heart failure not taking digitalis. *The American Journal of Cardiology.* 75 (8), 596-600.
4. Bussman, W., Storger, H., Hadler, D., Reifart, N., Fassbinder, W., Jungmann, E. & Kaltenbach, M. (1987) Long-term treatment of severe chronic heart failure with captopril: a double-blind, randomized, placebo-controlled, long-term study.. *Journal of Cardiovascular Pharmacology.* 9 (Supple 2), S50-S60.
5. Cohn J, Hawkins M, Levine H, and the Captopril-Digoxin Multicenter Research Group. (1988) Comparative effects of therapy with captopril and digoxin in patients with mild to moderate heart failure. *Jama.* 259 (4), 539-544.
6. Newman, T. J., Maskin, C. S., Dennick, L. G., Meyer, J. H., Hallows, B. G. & Cooper, W. H. (1988) The Renin-Angiotensin System and the Heart Effects of captopril on survival in patients with heart failure. *The American Journal of Medicine.* 84 (3), 140-144.
7. Cleland, J. G., Dargie, H. J., Ball, S. G., Gillen, G., Hodsman, G. P., Morton, J. J., East, B. W., Robertson, I., Ford, I. & Robertson, J. I. (1985) Effects of enalapril in heart failure: a double blind study of effects on exercise performance, renal function, hormones, and metabolic state. *British Heart Journal.* 54 (3), 305-312.
8. The CONSENSUS trial study group. (1987) Effects of Enalapril on Mortality in Severe Congestive Heart Failure. *N Engl J Med.* 316 (23), 1429-1435.
9. Drexler, H., Banhardt, U., Meinertz, T., Wollschläger, H., Lehmann, M. & Just, H. (1989) Contrasting peripheral short-term and long-term effects of converting enzyme inhibition in patients with congestive heart failure. A double-blind, placebo-controlled trial. *Circulation.* 79 (3), 491-502.
10. Dickstein, K., Barvik, S. & Aarsland, T. (1991) Effect of long-term enalapril therapy on cardiopulmonary exercise performance in men with mild heart failure and previous myocardial infarction. *JACC, Journal of American College of Cardiology.* 18596-602.
11. Dössegger, L., Aldor, E., Baird, M. G., Braun, S., Cleland, J. G. F., Donaldson, R., Jansen, L. J., Joy, M. D., Marin-Neto, J. A., Nogueira, E., Stahnke, P. L. & Storm, T. (1993) Influence of angiotensin converting enzyme inhibition on exercise performance and clinical symptoms in chronic heart failure: a multicentre, double-blind, placebo-controlled trial. *European Heart Journal.* 14 (suppl C), 18-23.
12. Enalapril CHF Investigators. (1987) Enalapril CHF Investigators Long-term effects of enalapril in patients with congestive heart failure: a multicenter, placebo-controlled trial. *Heart Failure.* 3102-107.
13. Erhardt, L., Maclean, A., Ilgenfritz, J., Gelperin, K. & Blumenthal, M. (1995) Fosinopril attenuates clinical deterioration and improves exercise tolerance in patients with heart failure. (Fosinopril Efficacy/Safety Trial (FEST) Study Group. *European Heart Journal.* 16 (12), 1892-1899.
14. Giles, T. & for Lisinopril Chronic Heart Failure Group. (1990) Lisinopril treatment of congestive heart failure-results of a placebo controlled trial. *Circulation.* 82 (Suppl 4), III-323-Abstract.
15. Gilbert, E. M., Sandoval, A., Larrabee, P., Renlund, D. G., O'Connell, J. B. & Bristow, M. R. (1993) Lisinopril lowers cardiac adrenergic drive and increases beta-receptor density in the failing human heart. *Circulation.* 88 (2), 472-480.
16. Gordon, M. (1991) *Evaluation of the Efficacy and Safety of Ramipril (HOE 498) in Patients With Congestive Heart Failure in a Placebo-Controlled Trial. Unpublished Report.* Somervilla, NJ, USA, Hoechst-Roussel Pharmaceuticals.
17. Gundersen, T., Swedberg, K., Amtorp, O., Remes, J. & Nilsson, B. (1994) Absence of effect on exercise capacity of 12-weeks treatment with ramipril in patients with moderate congestive heart failure. *European Heart Journal.* 15 (12), 1659-1665.
18. Hampton, J. R., Cowley, A. J. & Wnuk-Wojnar, A. M. (1998) Failure of an ACE inhibitor to improve exercise tolerance. A randomized study of trandolapril. *European Heart Journal.* 19 (12), 1823-1828.
19. Kleber, F. X., Niemöller, L. & Doering, W. (1992) Impact of converting enzyme inhibition on progression of chronic heart failure: results of the Munich Mild Heart Failure Trial. *British Heart Journal.* 67 (4), 289-296.
20. Lechat, P., Garnham, S. P., Desche, P. & Bounhoure, J. (1993) Contribution of ACE Inhibition to the Management of Heart Failure Efficacy and acceptability of perindopril in mild to moderate chronic congestive heart failure. *American Heart Journal.* 126 (3), 798-806.
21. Lemarie, J. (1992) *Multicenter Double-Blind Placebo Controlled Study of the Efficacy and Safety of Ramipril Administered Orally for 24 Weeks in the Treatment of Stable Chronic Congestive Cardiac Failure. Unpublished Report.* Paris, France, Laboratories Hoechst.
22. Lewis, G. & for International Study Group. (1989) Lewis GR Comparison of lisinopril versus placebo for congestive heart failure. *American Journal of Cardiology.* Feb 21 (63), 12D-16D.
23. Maass, L. (1991) *Double-Blind Comparative Trial With Ramipril and Placebo in Patients With Heart Failure (NYHA Class III-IV) Stabilized on Digitalis and Furosemides. Unpublished report.* Frankfurt, Germany, Hoechst Aktiengesellschaft.
24. Maass, L. (1991) *Efficacy and Safety of Ramipril (HOE498) in Patients With Congestive Heart Failure in a Double Blind Placebo Controlled Trial. Unpublished Report.* Frankfurt, Germany, Hoechst Aktiengesellschaft.
25. Maass, L. (1991) *Evaluation of the Effect of Ramipril (HOE 498) on Exercise Duration, Invasive Cardiac Hemodynamics Profiles, and Safety in Patients With Congestive Heart Failure. Unpublished Report.* Frankfurt, Germany, Hoechst Aktiengesellschaft.
26. McGarry, R. (1991) *Randomized, Double Blind, Multicenter Study Comparing Benazepril to Digoxin and to Placebo as Add On Therapy to Diuretic in Patients With CHF, NYHA Class II-III During a 12-Week Treatment Period, GHBA-194.* Unpublished report edition. Summit, NJ, Ciba-Geigy Pharmaceuticals.
27. McGrath, B. P., Arnolda, L., Matthews, P. G., Jackson, B., Jennings, G., Kiat, H. & Johnston, C. I. (1985) Controlled trial of enalapril in congestive cardiac failure. *British Heart Journal.* 54 (4), 405-414.
28. Magnani, B. & Mageli, C. (1986) Captopril in mild heart failure: preliminary observations of a long-term, double-blind, placebo-controlled multicentre trial. *Postgraduate Medical Journal.* 62 (Suppl 1), 153-158.
29. Northridge, D., Rose, E., Raffery, E., Elder, A., Shaw, T., Henderson, E. & Dargie, H. (1993) A multicentre, double-blind, placebo-controlled trial of quinapril in mild, chronic heart failure.  . *European Journal of Heart Failure.* Mar;14 (3), 403-409.
30. Reigger, G. (1990) The effects of ACE inhibitors on exercise capacity in the treatment of congestive heart failure. *Journal of Cardiovascular Pharmacology.* 15 (Suppl 2), 41-46.
31. Rucinska, E. (1991) *A double-blind placebo-controlled study to evaluate the effects of Enalapril in patients with chronic heart failure. Unpublished Report.* West Point, PA, Merck Sharpe & Dohme Research Laboratories.
32. Rucinska, E. (1991) *Enalapril vs. placebo in previously untreated patients with CHF. Unpublished Report.* West Point, Pa, Merck Sharpe & Dohme Research Laboratories.
33. SOLVD Investigators. (1991) Effect of Enalapril on Survival in Patients with Reduced Left Ventricular Ejection Fractions and Congestive Heart Failure. *N Engl J Med.* 325 (5), 293-302.
34. Sharpe, D. N., Murphy, J., Coxon, R. & Hannan, S. F. (1984) Enalapril in patients with chronic heart failure: a placebo-controlled, randomized, double-blind study. *Circulation.* 70 (2), 271-278.
35. Uprichard, A. (1994) *A 16-week double blind, placebo-randomized placebo controlled multicenter trial to evaluate the effects of withdrawal of Quinalapril Hydrochloride on exercise tolerance in patients with mild to moderate congestive heart failure.* Ann Arbor, Michigan, Parke-Davis Pharmaceutical Research.
36. Uprichard, A. (1994) *An 18-week double blind, optional titration, multicenter study to compare the efficacy and safety of orally administered Quinalapril Hydrochloride with Captopril and placebo in patients with congestive heart failure. Unpublished Report.* Ann Arbor, Michigan, Parke-Davis Pharmaceutical Research.
37. Uprichard, A. (1994) *A 12-week double-blind, placebo controlled study to determine the efficacy and safety of orally administered Quinalapril Hydrochloride in patients with congestive heart failure. Unpublished Report.* Ann Arbor, Michigan, Parke-Davis Pharmaceutical Research.
38. Zwehl, W., Rucinska, E. & for Lisinopril Chronic Heart Failure Investigators. (1990) Long-term effects of lisinopril in patients with chronic heart failure: a multicenter, placebo-controlled trial. In: Nicholls, M. (ed.). *A Focus on the Clinical Effects of a Long Acting ACE-Inhibitors/Heart Failure.* New York, NY, Raven Press. pp. 31-40

**Beta-blocker randomized control trials**

1. Australia/New Zealand Heart Failure Research Collaborative Group. (1997) Randomized, placebo-controlled trial of Carvedilol in patients with congestive heart failure due to ischaemic heart disease. *The Lancet.* 349 (9049), 375-380.
2. Beta-Blocker Evaluation of Survival Trial (BEST) Investigators. (2001) A trial of the beta-blocker Bucindolol in patients with advanced chronic heart failure. *New England Journal of Medicine.* 344:1659-1657.
3. Bristow, M. R., O'Connell, J. B., Gilbert, E. M., French, W. J., Leatherman, G., Kantrowitz, N. E., Orie, J., Smucker, M. L., Marshall, G. & Kelly, P. (1994) Dose-response of chronic beta-blocker treatment in heart failure from either idiopathic dilated or ischemic cardiomyopathy. Bucindolol Investigators. Circulation. 89 (4), 1632-1642.
4. Cohn, J. N., Fowler, M. B., Bristow, M. R., Colucci, W. S., Gilbert, E. M., Kinhal, V., Krueger, S. K., Lejemtel, T., Narahara, K. A., Packer, M., Young, S. T., Holcslaw, T. L. & Lukas, M. A. (1997) Safety and efficacy of carvedilol in severe heart failure. *Journal of Cardiac Failure.* 3 (3), 173-179.
5. Cleland, J., Pennell, D., Ray, S., Coats, A., Macfarlane, P., Murray, G., Mule, J. D., Vered, Z. & Lahiri, A. (2003) Myocardial viability as a determinant of the ejection fraction response to carvedilol in patients with heart failure (CHRISTMAS trial): randomized controlled trial. *The Lancet.* 362 (9377), 14-21.
6. CIBIS Investigators and Committees. (1994) A randomized trial of beta-blockade in heart failure: The Cardiac Insufficiency Bisoprolol Study (CIBIS). *Circulation.* 90 (4), 1765-1773.
7. CIBIS-II Investigators and Committees. (1999) The Cardiac Insufficiency Bisoprolol Study II (CIBIS-II): a randomized trial. *The Lancet.* 353 (9146), 9-13.
8. Colucci, W. S., Packer, M., Bristow, M. R., Gilbert, E. M., Cohn, J. N., Fowler, M. B., Krueger, S. K., Hershberger, R., Uretsky, B. F., Bowers, J. A., Sackner-Bernstein, J. D., Young, S. T., Holcslaw, T. L., Lukas, M. A. & for the US Carvedilol Heart Failure Study Group*. (1996) Carvedilol Inhibits Clinical Progression in Patients With Mild Symptoms of Heart Failure. *Circulation.* 94 (11), 2800-2806.
9. Packer, M., Coats, A., Fowler, M., Katus, H., Krum, H., Mohacsi, P., Rouleau, J., Tendera, M., Castaigne, A., Roecker, E., Schultz, M., Staiger, C., Curtin, E., DeMets, D. & for the Carvedilol Prospective Randomized Cumulative Survival Study (COPERNICUS) Group. (2001) Effect of Carvedilol on Survival in Severe Chronic Heart Failure. *N Engl J Med.* 344 (22), 1651-1658.
10. Edes, I., Gasior, Z. & Wita, K. (2005) Effects of nebivolol on left ventricular function in elderly patients with chronic heart failure: results of the ENECA study. *European Journal of Heart Failure.* 7 (4), 631-639.
11. Krum, H., Sackner-Bernstein, J. D., Goldsmith, R. L., Kukin, M. L., Schwartz, B., Penn, J., Medina, N., Yushak, M., Horn, E., Katz, S. D., Levin, H. R., Neuberg, G. W., DeLong, G. & Packer, M. (1995) Double-Blind, Placebo-Controlled Study of the Long-term Efficacy of Carvedilol in Patients With Severe Chronic Heart Failure. *Circulation.* 92 (6), 1499-1506.
12. Hjalmarson, Å., Goldstein, S., Fagerberg, B., Wedel, H., Waagstein, F., Kjekshus, J., Wikstrand, J., El Allaf, D., Vitovec, J., Aldershvile, J., Halinen, M., Dietz, R., Neuhaus, K., Janosi, A., Thorgiersson, G., Dunselman, P., Gullestad, L., Kuch, J., Herlitz, J., Rickenbacker, P., Ball, S., Gottlieb, S., Deedwania, P. & for the MERIT-HF study group. (2000) Effects of controlled-release metoprolol on total mortality, hospitalizations, and well-being in patients with heart failure: The metoprolol cr/xl randomized intervention trial in congestive heart failure (MERIT-HF). *Jama.* 283 (10), 1295-1302.
13. Wikstrand, J., Hjalmarson, Å. k., Waagstein, F., Fagerberg, B. j., Goldstein, S., Kjekshus, J., Wedel, H. & for the MERIT-HF study group. (2002) Dose of metoprolol CR/XL and clinical outcomes in patients with heart failure: Analysis of the experience in metoprolol CR/XL randomized intervention trial in chronic heart failure (MERIT-HF). *Journal of the American College of Cardiology.* 40 (3), 491-498.
14. Bristow, M. R., Gilbert, E. M., Abraham, W. T., Adams, K. F., Fowler, M. B., Hershberger, R. E., Kubo, S. H., Narahara, K. A., Ingersoll, H., Krueger, S., Young, S., Shusterman, N. & for the MOCHA Investigators. (1996) Carvedilol Produces Dose-Related Improvements in Left Ventricular Function and Survival in Subjects With Chronic Heart Failure. Circulation. 94 (11), 2807-2816.
15. Olsen, S. L., Gilbert, E. M., Renlund, D. G., Taylor, D. O., Yanowitz, F. D. & Bristow, M. R. (1995) Carvedilol improves left ventricular function and symptoms in chronic heart failure: A double-blind randomized study. *Journal of the American College of Cardiology.* 25 (6), 1225-1231.
16. Packer, M., Bristow, M. R., Cohn, J. N., Colucci, W. S., Fowler, M. B., Gilbert, E. M. & Shusterman, N. H. (1996) The Effect of Carvedilol on Morbidity and Mortality in Patients with Chronic Heart Failure. *N Engl J Med.* 334 (21), 1349-1355.
17. Packer, M., Colucci, W. S., Sackner-Bernstein, J. D., Liang, C., Goldscher, D. A., Freeman, I., Kukin, M. L., Kinhal, V., Udelson, J. E., Klapholz, M., Gottlieb, S. S., Pearle, D., Cody, R. J., Gregory, J. J., Kantrowitz, N. E., LeJemtel, T. H., Young, S. T., Lukas, M. A., Shusterman, N. H. & for the PRECISE Study Group*. (1996) Double-Blind, Placebo-Controlled Study of the Effects of Carvedilol in Patients With Moderate to Severe Heart Failure: The PRECISE Trial. *Circulation.* 94 (11), 2793-2799.
18. The RESOLVD Investigators. (2000) Effects of Metoprolol CR in Patients With Ischemic and Dilated Cardiomyopathy: The Randomized Evaluation of Strategies for Left Ventricular Dysfunction Pilot Study. *Circulation.* 101 (4), 378-384.
19. Flather, M. D., Shibata, M. C., Coats, A. J. S., Van Veldhuisen, D. J., Parkhomenko, A., Borbola, J., Cohen-Solal, A., Dumitrascu, D., Ferrari, R., Lechat, P., Soler-Soler, J., Tavazzi, L., Spinarova, L., Toman, J., Böhm, M., Anker, S. D., Thompson, S. G. & Poole-Wilson, P. A. (2005) Randomized trial to determine the effect of nebivolol on mortality and cardiovascular hospital admission in elderly patients with heart failure (SENIORS). *European Heart Journal.* 26 (3), 215-225.
20. Sturm, B., Pacher, R., Strametz-Juranek, J., Berger, R., Frey, B. & Stanek, B. (2000) Effect of B-blockade with atenolol on progression of heart failure in patients pretreated with high-dose enalapril. *European Journal of Heart Failure.* 2 (4), 407-412.
21. Waagstein, F., Strömblad, O., Andersson, B., Böhm, M., Darius, M., Delius, W., Goss, F., Osterziel, K. J., Sigmund, M., Trenkwalder, S. -. & Wahlqvist, I. (2003) Increased exercise ejection fraction and reversed remodelling after long-term treatment with metoprolol in congestive heart failure: a randomized, stratified, double-blind, placebo-controlled trial in mild to moderate heart failure due to ischemic or idiopathic dilated cardiomyopathy. *European Journal of Heart Failure.* 5 (5), 679-691.
22. Wisenbaugh, T., Katz, I., Davis, J., Essop, R., Skoularigis, J., Middlemost, S., Röthlisberger, C., Skudicky, D. & Sareli, P. (1993) Long-term (3-month) effects of a new beta-blocker (nebivolol) on cardiac performance in dilated cardiomyopathy. *Journal of the American College of Cardiology.* 21 (5), 1094-1100.

**Aldosterone antagonist randomized control trials**

1. Agostoni, P., Magini, A., Andreini, D., Contini, M., Apostolo, A., Bussotti, M., Cattadori, G. & Palermo, P. (2004) Spironolactone improves lung diffusion in chronic heart failure. *European Heart Journal.* 26 (2), 159-164.
2. Boccanelli, A., Cacciatore, G., Mureddu, G. F., de Simone, G., Clemenza, F., De Maria, R., Di Lenarda, A., Gavazzi, A., Latini, R., Masson, S., Porcu, M., Vanasia, M., Gonzini, L., Maggioni, A. P. & Investigators.,on behalf of the AREA IN-CHF. (2007) Baseline characteristics of patients recruited in the AREA IN-CHF study (Antiremodelling Effect of Aldosterone Receptors Blockade with Canrenone in Mild Chronic Heart Failure). *Journal of Cardiovascular Medicine.* 8 (9), 683-691.
3. Barr, C. S., Lang, C. C., Hanson, J., Arnott, M., Kennedy, N. & Struthers, A. D. Effects of adding *spironolactone* to an angiotensin-converting enzyme inhibitor in chronic congestive heart failure secondary to coronary artery disease. *American Journal of Cardiology.* 76 (17), 1259-1265.
4. Berry, C., Murphy, N., De Vito, G., Galloway, S., Seed, A., Fisher, C., Sattar, N., Vallance, P., Hillis, W. S. & McMurray, J. (2007) Effects of aldosterone receptor blockade in patients with mild/moderate heart failure taking a beta-blocker. *European Journal of Heart Failure.* 9 (4), 429-434.
5. Chan, A. K. Y., Sanderson, J. E., Wang, T., Lam, W., Yip, G., Wang, M., Lam, Y., Zhang, Y., Yeung, L., Wu, E. B., Chan, W. W. M., Wong, J. T. H., So, N. & Yu, C. (2007) Aldosterone Receptor Antagonism Induces Reverse Remodeling When Added to Angiotensin Receptor Blockade in Chronic Heart Failure. *Journal of the American College of Cardiology.* 50 (7), 591-596.
6. Gao, X., Peng, L., Adhikari, C. M., Lin, J. & Zuo, Z. (2007) Spironolactone Reduced Arrhythmia and Maintained Magnesium Homeostasis in Patients With Congestive Heart Failure. *Journal of Cardiac Failure.* 13 (3), 170-177.
7. The RALES Investigators. (1996) Effectiveness of Spironolactone Added to an Angiotensin-Converting Enzyme Inhibitor and a Loop Diuretic for Severe Chronic Congestive Heart Failure (The Randomized Aldactone Evaluation Study [RALES]). *The American Journal of Cardiology.* 78 (8), 902-907.
8. Pitt, B. & Roniker, B. (1999) Eplerenone, a novel selective aldosterone receptor antagonist (SARA): dose finding study in patients with heart failure. *Journal of the American College of Cardiology.* 15 (Suppl 1), 188A.
9. Pitt, B., Zannad, F., Remme, W. J., Cody, R., Castaigne, A., Perez, A., Palensky, J. & Wittes, J. (1999) The Effect of Spironolactone on Morbidity and Mortality in Patients with Severe Heart Failure. *N Engl J Med.* 341 (10), 709-717.
10. Inspra. (2004) *Study 402. Inspra Drug Approval Package.* Drug approval package provided by manufacturer edition. Food and Drug Administration.
11. Tsutamoto, T., Wada, A., Maeda, K., Mabuchi, N., Hayashi, M., Tsutsui, T., Ohnishi, M., Sawaki, M., Fujii, M., Matsumoto, T., Matsui, T. & Kinoshita, M. (2001) Effect of spironolactone on plasma brain natriuretic peptide and left ventricular remodeling in patients with congestive heart failure. *Journal of the American College of Cardiology.* 37 (5), 1228-1233.
12. Zannad, F., McMurray, J. J. V., Krum, H., van Veldhuisen, D. J., Swedberg, K., Shi, H., Vincent, J., Pocock, S. J. & Pitt, B. (2011) Eplerenone in Patients with Systolic Heart Failure and Mild Symptoms. *N Engl J Med.* 364: 11-21
